# The correlations between kinematic profiles and cerebral hemodynamics suggest changes of motor coordination in single and bilateral finger movement

**DOI:** 10.3389/fnhum.2022.957364

**Published:** 2022-08-18

**Authors:** Guangquan Zhou, Yuzhao Chen, Xiaohan Wang, Hao Wei, Qinghua Huang, Le Li

**Affiliations:** ^1^School of Biological Science and Medical Engineering, Southeast University, Nanjing, China; ^2^Institute of Medical Research, Northwestern Polytechnical University, Xi’an, China; ^3^School of Artificial Intelligence, OPtics and ElectroNics (iOPEN), Northwestern Polytechnical University, Xi’an, China

**Keywords:** fNRIS, brain network, finger movement, motor coordination, in-phase, anti-phase

## Abstract

**Objective:**

The correlation between the performance of coordination movement and brain activity is still not fully understood. The current study aimed to identify activated brain regions and brain network connectivity changes for several coordinated finger movements with different difficulty levels and to correlate the brain hemodynamics and connectivity with kinematic performance.

**Methods:**

Twenty-one right-dominant-handed subjects were recruited and asked to complete circular motions of single and bilateral fingers in the same direction (in-phase, IP) and in opposite directions (anti-phase, AP) on a plane. Kinematic data including radius and angular velocity at each task and synchronized blood oxygen concentration data using functional near-infrared spectroscopy (fNIRS) were recorded covering six brain regions including the prefrontal cortex, motor cortex, and occipital lobes. A general linear model was used to locate activated brain regions, and changes compared with baseline in blood oxygen concentration were used to evaluate the degree of brain region activation. Small-world properties, clustering coefficients, and efficiency were used to measure information interaction in brain activity during the movement.

**Result:**

It was found that the radius error of the dominant hand was significantly lower than that of the non-dominant hand (*p* < 0.001) in both clockwise and counterclockwise movements. The fNIRS results confirmed that the contralateral brain region was activated during single finger movement and the dominant motor area was activated in IP movement, while both motor areas were activated simultaneously in AP movement. The Δhbo were weakly correlated with radius errors (*p* = 0.002). Brain information interaction in IP movement was significantly larger than that from AP movement in the brain network (*p* < 0.02) in the right prefrontal cortex. Brain activity in the right motor cortex reduces motor performance (*p* < 0.001), while the right prefrontal cortex region promotes it (*p* < 0.05).

**Conclusion:**

Our results suggest there was a significant correlation between motion performance and brain activation level, as well as between motion deviation and brain functional connectivity. The findings may provide a basis for further exploration of the operation of complex brain networks.

## Introduction

Coordination ability is defined as the ability of both limbs to perform a specific movement through the coordination of the nervous system and muscles, which is essential in daily life (Walhain et al., 2016). For instance, daily tasks, such as eating, dressing, and using tools, require the coordinated movement of multiple muscles in the upper body (Yu et al., 2017), often consisting of the symmetrical way of the hands (such as serving a plate) or asymmetrical way (such as opening a can). Previous studies have revealed that coordination ability training evokes brain activation that may contribute to the reorganization of the motor cortex (Li et al., 2013; Han and Kim, 2016; Wu et al., 2020). Recently, it has been demonstrated that bilateral coordinated movement can promote the neurotic recombination of the motor region of patients after stroke (Wu et al., 2020) and benefit stroke patients’ recovery (Fu et al., 2021). However, the underlying neural mechanisms remain unclear in coordination ability training. Therefore, it is still essential to further explore the information related to the neural mechanism of motor control in the coordination movement.

The bilateral coordinated movements can be modeled as a combination of anti-phase (AP) and in-phase (IP) movements (Shih et al., 2021). The bilateral IP movements contract homologous muscles on both sides simultaneously, while the AP movements alternately activate the corresponding bilateral muscles (Swinnen, 2002). Previous research suggests that IP movements depend more on the left-dominant cerebral cortical control, that is, the dominant hemisphere (Maki et al., 2008). In contrast, both dominant and non-dominant hemispheres all contribute to the control of the AP movements (Shih et al., 2021). Moreover, previous Transcranial Magnetic Stimulation (TMS) study reveals that the movement-related facilitation from the right front motor cortex to the left primary motor cortex (rPMd-IM1) is correlated to a better performance in AP movement (Liuzzi et al., 2011), suggesting that efficient brain connectivity is essential in bilateral coordinated movements. However, most previous studies have only focused on the function of specific brain regions in coordinated movement, ignoring the mutual influence and connections of various brain regions on bilateral coordinated movements.

Previous electroencephalogram (EEG) studies disclose age-related differences inbilateral coordinated movements at the neural activity level and brain hemisphere connectivity (Shih et al., 2021). However, there is only the analysis of the whole brain activity due to the spatial resolution of EEG when performing bilateral coordinated movements, still lacking functional analysis of specific brain regions and their connectivity. Brain imaging techniques have also examined brain region activity analysis during coordinated movement. For example, Heuninckx et al. (2008) disclosed that coordinated movement is highly correlated with the activation of three regions: classical motor coordination, higher-level sensorimotor, and frontal regions. Moreover, in a functional magnetic resonance imaging (fMRI) study, networks constructed by temporal correlations in the finger-tapping task explain the functional impact of brain regions, revealing the contributions of these regions to the coordinated movements (Maki et al., 2008). Nevertheless, these researches neglect the brain connectivity activity under functionally coordinated movements. As a result, it remains unclear whether the connections between the cerebral cortex are related to motor performance during coordinate movements.

Compared to EEG, functional near-infrared spectroscopy (fNIRS) has advantages in functional connection analysis with higher probe resolution in space (Ge et al., 2017). By contrast, although fMRI can provide high-precision scans of the brain, the technology of limitations of the equipment results in the experiment design merely covering elementary movements, such as finger tapping tasks (Zhuang et al., 2005). Furthermore, fNIRS is more resistant to motion artifacts and electroless noise than EEG and fMRI. As a non-invasive neuroimaging approach, fNIRS could measure the changes in hemoglobin and deoxygenated hemoglobin concentrations, thus indicating the degree of brain activation (Toronov et al., 2000; Cui et al., 2011; Ferrari and Quaresima, 2012; Sukal-Moulton et al., 2018). On the other hand, fNIRS is also a harmless and low-cost approach for detecting brain activity, which provides a more temporal resolution of higher blood motivational signals (Strangman et al., 2002). fNIRS has emerged as a promising field to study coordination capabilities using its real-time multi-channel blood oxygen concentration signals, especially for the function and connections analysis of various brain regions. Therefore, in the present study, we utilized fNIRS to investigate the mutual influence of corresponding active brain regions during bilateral coordinated movements, which might benefit to understand the underlying neural mechanisms in coordination ability training. We hypothesized that the changes in brain activity correlate with the coordinate ability, and the connections between the cerebral cortex during coordinate movements are related to the motor performance of limb movement. The relationship between the kinematics and the information transmit ability of the brain network was also scrutinized by analyzing the correlation values of the kinematic data and the network indicators. In addition, we also adopt the graph theory for characterizing complex networks of the brain (Bullmore and Sporns, 2009) along with several topological aspects (Rubinov and Sporns, 2010).

## Materials and methods

### Participants

Twenty-one healthy volunteers (11 males and 10 females, age: 24.62 ± 1.5 years) were recruited from the Northwestern Polytechnical University to participate in the study. According to the Edinburgh Handedness Inventory, all subjects are right-handed dominant with normal vision and without a history of epilepsy or other psychiatric episodes. The institutional ethical committee approved this study, which was registered on the Chinese Clinical Trial Registry (ChiCTR2200057839). All subjects have understood the purpose and content of the experiment and signed informed consent before participation in the experiment. Before the formal experiment, the right-hand preliminary experimentation was performed to ensure that the brain area activated normally. Also, the subjects were allowed to have enough rest time to ensure their concentration during the experiment.

### Experimental procedures

#### Circle drawing task

**FIGURE 1:**
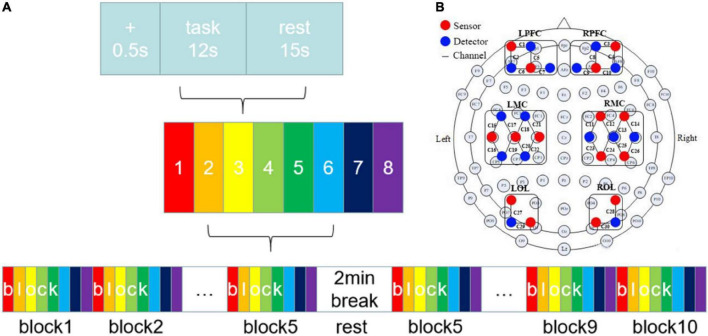
**(A)** Experiment procedure. There were eight tasks in this study. **(B)** The layout of fNIRS channels. The red dots represented sources and the blue dots represented detectors. The 15 sources and 15 detectors constitute 30 channels, overlaying six brain regions: LPFC, RPFC, LMC, RMC, LOL, and ROL.

**FIGURE 2:**
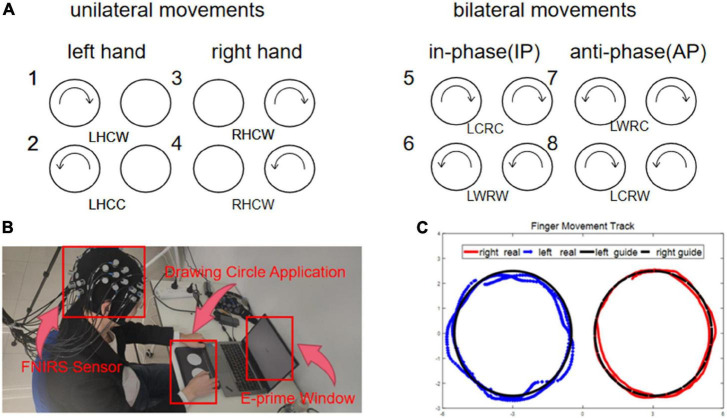
**(A)** Condition 1–2: left side tasks, condition 3–4: right side tasks, condition 5–6: anti-phase tasks, condition 7–8: in-phase tasks. **(B)** Position 1: hat with sensors, position 2: the application interface of collecting finger movement trajectory, position 3: the interface of E-prime. **(C)** The data of motion track. (1) the blue line was the real trajectory of left hand, (2) the red line was the real trajectory of right hand, and (3) the black line was the guide trajectory.

The subjects needed to complete eight clockwise and counterclockwise finger circle movements, categorized into unilateral and bilateral coordination movements. The bilateral coordination movements were further divided into anti-phase (AP) and in-phase (IP) movements. Before the formal experiment, all subjects took the same trial training as the formal experiment without wearing the near-infrared collection cap to facilitate adaptation to the movement speed and the laboratory environment. Figure 1A shows the experimental procedure. Before the formal experiment, all subjects took the same trial training as the formal experiment without wearing the near-infrared collection cap to facilitate adaptation to the movement speed and the laboratory environment. At the beginning of the experiment, the subjects were instructed to pay attention to the experiment guidance using a sound and a sign prompt. Then, they followed the movement mode prompting on the monitor during the experiment with two semi-circular arrows, which were randomly assigned from the eight kinds of movements (Figure 2A). Also, the subjects need to confirm the circle drawing mode using the self-designed circle drawing assistant software mode and E-Prime3.0 (Psychology Software Tools, Pittsburgh, PA, United States) shown in Figure 2B to start the fNIRS and kinematic data collection. The subjects performed each movement for approximately 12 s. The examination was repeated to complete all eight movements composing a block unit with a rest of 15 s between two consequent movement trials. In addition, each subject was required to conduct 10-block units with a rest of 2 min after completing five-block units.

#### Data acquisition

The fNIRS signal with the wavelengths of near-infrared light at 740, 808, and 850 mm was collected using a multi-channel fNIRS system (Danyang Huichuang Medical Instrument Co., Ltd., China) with a sampling rate of 11 Hz. As shown in Figure 1B, we employed the standard cap with an international 10–20 system as the reference for the near-infrared probe layout. Also, the light source was spaced at 30 mm from the probe to ensure signal detection from the brain’s gray matter. Finally, a total of 30 channels using the midpoint of the detector cover the left and right prefrontal cortex (LPFC, RPFC), the left and right motor cortex (LMC, RMC), and the left and right occipital lobes (LOL, ROL). In addition, the hair underneath the cap was carefully pulled aside to allow complete contact between the probe and the scalp, thus ensuring signal strength and effectiveness. The participants’ head was covered with a spring mounted to secure the light source and probe. On the other hand, as illustrated in Figure 2B, we developed a self-designed application using Unity3D to collect the kinematic data at a frequency of 28.5 Hz, which captures the circle trajectory (Figure 2C) of the subject’s finger on the tablet for further analysis. The self-designed application is also developed to guide different circle movements at various speeds.

### Data analysis

#### Data preprocess

In this experiment, we aim to explore the relationship between the nervous system activity and motor control ability. Therefore, we first removed the redundant data and motion artifacts from the kinematic and fNIRS data for the following analysis. The redundant kinematic data generated by mode switching was removed by preprocessing the kinematic data, followed by calculating the standard deviation and error of radius and angular velocity to evaluate the motor control ability of the participants. Because the task is circle movements, kinematic data were required to convert to polar coordinates from Cartesian coordinates. The standard deviation and error of the radius and the angular velocity in polar coordinates from the recorded kinematic data were calculated and analyzed in MATLAB (The Mathworks, United States). By contrast, the blood oxygen signal included two change signals, oxyhemoglobin (Δhbo) and deoxyhemoglobin (Δhbr) concentration signals. According to the Beer–Lambert law, the blood oxygen concentration could be calculated from light intensity change under the assumption of constant scattering (Boas et al., 2004). Similarly, we employed a sliding averaging window with a 10-point duration on the fNIRS data to automatically identify the motion artifacts caused by the relative movement of the electrode and the skin using the moving standard deviation larger than 5 mmol/L and manual refinements, followed by the cubic spline interpolation to artifacts removal. Moreover, a band-pass filter of 0.021–0.6 Hz and a band-stop filter of 0.08–0.12 Hz were applied to remove physiological signal interferences, including pulse oscillations of about 1 Hz caused by heartbeat, 0.2–0.3 Hz disturbances caused by respiratory activities, Mayer waves generated by blood pressure oscillations in the range of 0.08–0.12 Hz, unexplained spontaneous low-frequency signals (0.06–0.1 Hz), and ultra-low frequency oscillation (0–0.04 Hz).

#### Kinematic data analysis

The kinematic profiles of the coordinate movement were revealed by the following parameters:

Radius standard deviation (SDR): The standard deviation of radius provides the variability of the trajectory within a trial by calculating the radius change rate of the trajectory in each trial. A lower standard deviation of the radius indicates a more stable trajectory during motion. It is defined as follows:


(1) $$\mathrm{SDR}=\sqrt{\frac{\sum_{i=1}^{\text{n}}{\left({\text{r}}_{\text{i}}-\bar{\text{r}}\right)}^{2}}{\text{n}}}$$

Radius Error (RE): The radius error provides the relative position error between the position of the tested circle and the standard circle in one trial by calculating the relative position distance in each trial, and it reflects the track performance of subjects in the movements. The lower the radius error, the participants have the better performance. It is defined as follows:


(2) $$\text{RE}=\sqrt{\frac{\sum_{i=1}^{\text{n}}{\left({\text{r}}_{\text{i}}-{\text{r}}_{\text{o}}\right)}^{2}}{\text{n}}}$$

Angular velocity standard deviation (SDAV): The standard deviation, in which the angular velocity deviated from the average value in the circle, reflects the fluctuation of the movement process. It is defined as:


(3) $$\text{SDAV}=\sqrt{\frac{\sum_{i=1}^{\text{n}}{\left({\text{w}}_{\text{i}}-\bar{\text{w}}\right)}^{2}}{\text{n}}}$$

Angular velocity error (AVE): The difference between the angular velocity and the standard angular velocity in the circle represents the following performance. It is defined as:


(4) $$\text{AVE}=\sqrt{\frac{\sum_{i=1}^{\text{n}}{\left({\text{w}}_{\text{i}}-{\text{w}}_{\text{o}}\right)}^{2}}{\text{n}}}$$

#### FNIRS data analysis

To identify the function of the brain region, the General Linear Model (GLM) (Friston et al., 1997) can first be used to analyze the hemodynamic response from the fNIRS data, thus scrutinizing brain activation regions and then certifying locations for brain network analysis. GLM is a commonly used method for the standard linear estimates of the hemodynamic response from fNIRS data, thus obtaining regions of the brain activation with a good temporal resolution (Barahimi et al., 2021). Also, the integral value of fNIRS data representing blood oxygen concentration can measure brain activity intensity during the task.

On the other hand, network analysis is necessary to further explore the relationship between the connections of the brain region after the brain activity analysis. Brain network connections can generally be divided into anatomical, functional, and effective connections (Lee et al., 2003), among which functional connectivity refers to the temporal correlation of spatially distant neurons due to physiological events. Previous studies have suggested that functional connectivity can benefit understanding brain connectivity activity under functionally coordinated movements (Sauseng and Klimesch, 2008; Liu et al., 2022). As Keles et al. (2016) have identified that there is a correlation between neural activity in the alpha and beta bands in the EEG and changes in hemoglobin concentration in fNIRS, we perform a similar activation analysis for fNIRS to validate the corresponding activation region for coordinated movements similar to previous EEG studies (Shih et al., 2021). Moreover, the phase lock value (PLV) is the common method to define non-directional functional connectivity (Mutlu et al., 2012; Wu et al., 2012), which can be used to construct a brain network for exploring the inter-relationship of each brain region, then further revealing the mechanism of brain activity to coordinate movements after further analysis of network indicators. Therefore, in the present brain network analysis, we employed wavelet transform to obtain the oxyhemoglobin concentration signal in different frequency bands (high-frequency band; 0.145–0.6 Hz and low-frequency band II; 0.021–0.145 Hz) (Shiogai et al., 2010), followed by calculating PLV between each channel from the instantaneous phase of the wavelet, thus constructing the functional connectivity matrix. Finally, we calculated commonly used network indicators, including the local and global efficiency and clustering coefficient, to characterize the functional connectivity of brain regions (Mukli et al., 2021), where the clustering coefficient is a measure of network isolation and global efficiency measures network integration. By contrast, local efficiency represents the ability to integrate adjacent nodes of a given node corresponding to a brain region (Bullmore and Sporns, 2009; Xu et al., 2022). Furthermore, the correlation between neural activity and control ability was obtained by calculating neural activity indicators and motor parameters.

##### Brain activation analysis

According to multiple regression analysis, the GLM was inserted into linear combinations of regressors to estimate the activation amplitudes correlated to specific movements (Wanniarachchi et al., 2020). Moreover, we derive the integral value of the cerebral blood oxygen concentration in the task state minus the concentration in the resting state, which defines the degree of brain activation in the task state relative to the resting state. The higher the integral value, the higher the activation intensity of this brain area.

##### Brain network analysis

Each brain region corresponding to a specific function forms a dynamic network with a complex distribution. Consequently, there is a dynamic interaction among each functional brain region to coordinate and respond to the specific environment and task requirements. Functional connectivity analysis is a valuable method to identify these brain region interconnections by calculating the correlation between the activity of neurons distributed in different spatial locations. In this study, the blood oxygen signal can be divided into two prominent frequency bands, including high-frequency band I (HF; 0.145–0.6 Hz) and low-frequency band II (LF; 0.021–0.145 Hz), among which the low-frequency band originated from the myogenic and neurogenic activity (Shiogai et al., 2010). Therefore, we extract these LF signals for the functional connectivity analysis to explore the brain functional interaction, disclosing the relationship between neural signals and kinematics.

Continuous wavelet transform (CWT) is the state-of-art time-frequency method to characterize the non-stationarity brain neurogenic activity signals. This study adopts the Morlet wavelet (Grinsted et al., 2004) to scrutinize the physiological signals in the LF band. At a specific frequency f and time point tn, the complex wavelet coefficients are derived as below:


(5) $$w_{k}\left(t_{n}\right)=W_{k}\left(f,t_{n}\right)\cdot e^{i\varphi_{k}\left(f,t_{n}\right)}=a_{k}\left(f,t_{n}\right)+ib_{k}\left(f,t_{n}\right)$$

*φ_k_(*f*,*t*_*n*_)*is the Morlet wavelet.

The wavelet instantaneous phase [k(f,t_n_)] is defined as:


(6) $$\varphi_{\text{k}}\left(f,{\text{t}}_{\text{n}}\right)={\text{arctan[b}}_{\text{k}}\left(f,{\text{t}}_{\text{n}}\right)/{\text{a}}_{\text{k}}\left(f,{\text{t}}_{\text{n}}\right)]$$

By the wavelet transform, we can extract the dynamic phase information from the different frequency bands of the time series to transform the time series to phase series for further channel-channel correlation analysis.

Some previous studies adopt the Pearson correlation analysis, the coherence coefficient, and the phase lock value for the network connection analysis (Wu et al., 2020), among which the phase lock value reflected the synchronization connectivity. The phase lock value not only represented the phase difference tendency of the overall signal but also provide more information interaction compare to correlation analysis and coherence coefficient. The stronger connection between the two channels, the larger the value of phase lock, and vice versa. Therefore, a diagonally symmetrical network is generally constructed by calculating the phase-locking value between the signals of each channel. As a result, the dynamic phase information of different frequency intervals in the LF band was extracted, followed by the subsequent phase-coupled signal model for brain region connection analysis.

The phase lock value is then defined as below:


(7) $$\text{PLV}=\left|{\text{n}}^{-1}\sum_{t=1}^{n}{\text{e}}^{i\left(\varphi \mathrm{xt}-\varphi \mathrm{yt}\right)}\right|$$

where φ*xt* and φ*yt* are the phase angle of the signals x and y in time t.

For the correlation matrix based on the phase lock value, the threshold was set under the condition of ensuring the connectivity of the brain network to remove weak connections. For brain network analysis, channels were defined as nodes and connections between channels were defined as edges according to the requirements of graph theory. According to the correlation matrix that constructed phase-value between these channels, the brain network could provide information on functional connectivity among the brain regions. For the brain functional network, global and local metrics were computed for each participant. Global metrics include global efficiency, clustering coefficient, and small-world properties (Watts and Strogatz, 1998; Latora and Marchiori, 2001; Onnela et al., 2005). Local metrics cover node degree, node efficiency, and node-local efficiency (Achard and Bullmore, 2007; Buckner et al., 2009; Liang et al., 2013; Liao et al., 2013, 2017). These global and local indicators were used to measure the ability of brain network connectivity. We used the Gretna toolbox (Rubinov and Sporns, 2010) to calculate these values.

##### Statistical analysis

Two-way analysis of variance (ANOVA) served as assessing between-group differences in handedness and orientation in unilateral movements. Three-way analysis of variance (ANOVA) served as assessing of handedness, orientation, and condition (anti-phase and in-phase) in bilateral movements. Independent *t*-tests were performed for each channel in the brain region and the results were false discovery rate (FDR) verified. Paired *t*-tests in 21 participants for small-world properties, global efficiency, clustering coefficient, node efficiency, and node-local efficiency in network analysis were performed. Pearson correlation was applied to test the correlation between network metrics and kinematic data and the correlation between Δhbo and kinematic data. The significance level was set at *P* < 0.05. The data analysis was performed with the software of IBM SPSS statistics 26 (IBM Inc., WA, United States) and MATLAB R2021a (The MathWorks, United States).

## Results

### Unilateral movements

**FIGURE 3:**
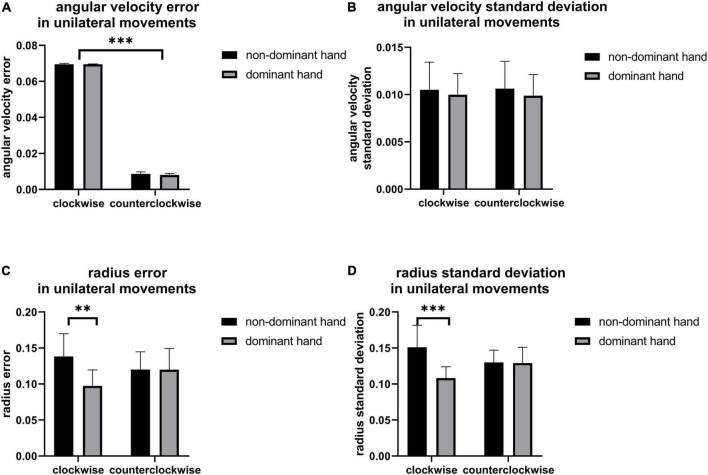
The **(A)** angular velocity error, **(B)** angular velocity standard deviation, **(C)** radius error, and **(D)** radius standard deviation of unilateral movement, the significant difference between groups is represented by ^**^(*p* < 0.01) and ^***^ (*p* < 0.001).

**FIGURE 4:**
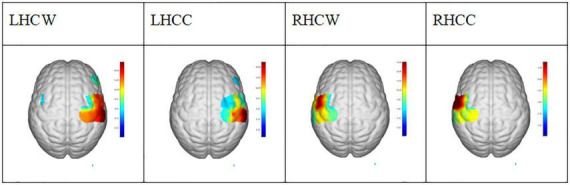
The activation region (*t* = 0.05) under general linear model in conditions of LHCW, LHCC, RHCW, and RHCC. LHCW, left hand clockwise. LHCC, left hand counterclockwise. RHCW, right hand clockwise. RHCC, right hand counterclockwise.

**FIGURE 5:**
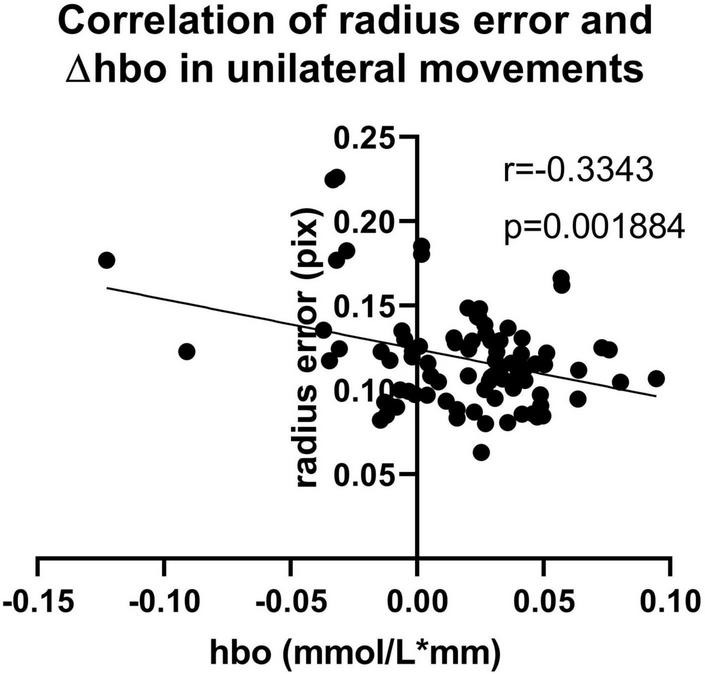
Correlation of radius error and Δhbo (corr = –0.335, *p* = 0.002) in unilateral movements.

Figure 3 shows the error and standard deviation of angular velocity, moving radius errors, and the standard deviation of moving radius in unilateral motions. It can be found that the average angular velocity along the clockwise direction is significantly higher than that of counterclockwise for the unilateral motion of single hands (*p* < 0.001, Figure 3A), implying clockwise movement is easier than counterclockwise movement. By contrast, there are no other differences found between the two moving directions (Figure 3A). Also, the moving radius errors (*p* = 0.002, Figure 3C) and the standard deviation of the moving radius (*p* < 0.001, Figure 3D) are significantly higher in the non-dominant hand. There are significant differences among the channels under the four unilateral movements (*p* < 0.05, Figure 4), suggesting the contralateral brain area activation. There is a weak negative correlation between radius error and cerebral cortical blood flow concentration changes (Figure 5) in unilateral movements (corr = −0.335, *p* = 0.002).

### Bilateral movements

**FIGURE 6:**
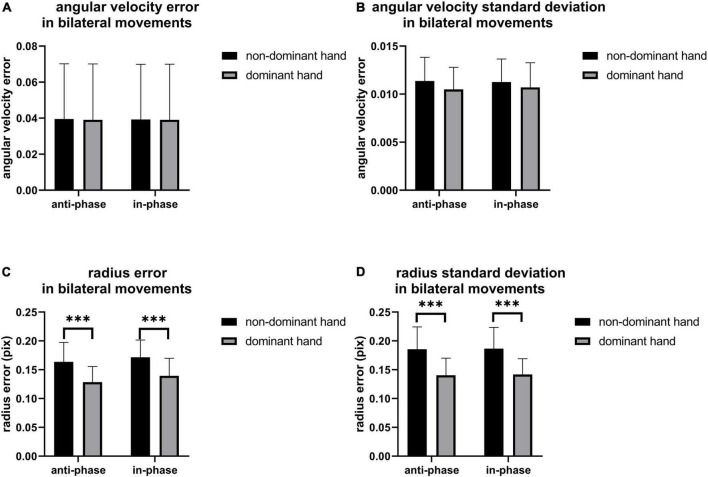
The **(A)** angular velocity error, **(B)** angular velocity standard deviation, **(C)** radius error and **(D)** radius standard deviation of bilateral movement, the significant difference between groups was represented by ***p < 0.001.

**FIGURE 7:**
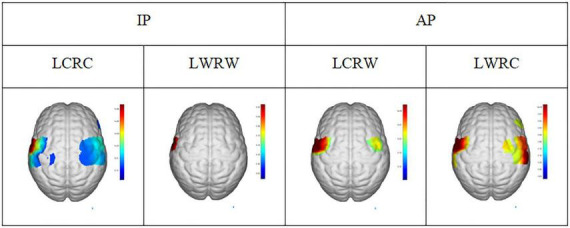
The activation region (*t* = 0.05) under general linear model in conditions of LCRC, LWRW, LCRW, and LWRC. LCRC, left hand clockwise and right hand clockwise. LWRW, left hand counterclockwise and right hand counterclockwise. LCRW, left hand clockwise and right hand counterclockwise. LWRC, left hand counterclockwise and right hand clockwise.

As shown in Figure 6, there are no statistical differences in angular velocity between IP and AP movements. The moving radius errors (Figure 6C) and the standard deviation of the moving radius (Figure 6D) of the non-dominant hand are significantly higher than the dominant hand in all bilateral motions (*p* < 0.001), implying that the non-dominant hand has worse performance. On the other hand, as illustrated in Figure 7, we observed apparent activation in the dominant hemisphere’s motor cortex (MC) when the subjects performed IP bilateral clockwise movements. There is significant motor cortex activation in the non-dominant hemisphere despite lower activation intensity than in the dominant hemisphere. However, brain activation in MC of non-dominant hemispheres is not evident during counterclockwise IP movements. By contrast, we found a similar significant activation distribution trend in the MC of both hemispheres during all AP movements.

### Brain network measurements

**FIGURE 8:**
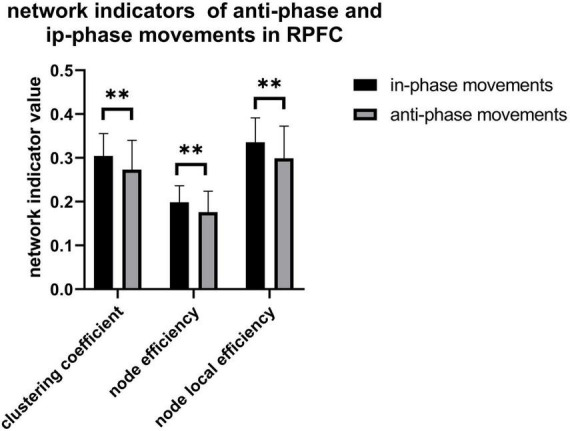
Clustering coefficient (*p* = 0.018), node efficiency (*p* = 0.019), and node-local efficiency (*p* = 0.012) in RPFC. ^**^p < 0.05.

**FIGURE 9:**
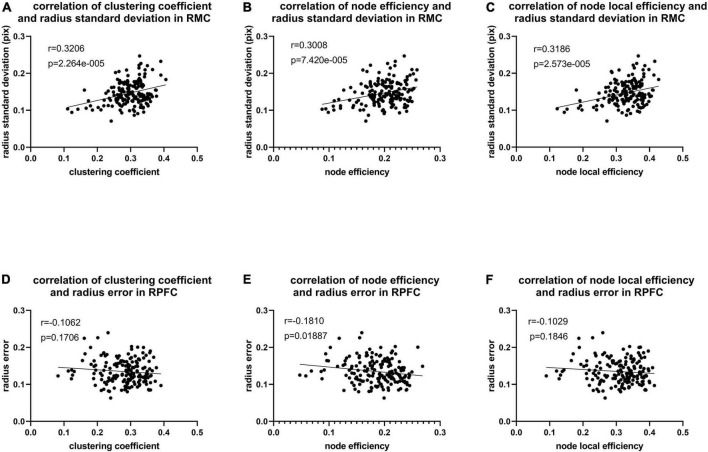
**(A)** Correlation of radius standard deviation and clustering coefficient (corr = 0.321, *p* < 0.001) in RMC. **(B)** Correlation of radius standard deviation and node efficiency (corr = 0.301, *p* < 0.001) in RMC. **(C)** Correlation of radius standard deviation and node local efficiency (corr = 0.319, *p* < 0.001) in RMC. **(D)** Correlation of radius error and clustering coefficient (corr = –0.106, *p* = 0.171) in RPFC. **(E)** Correlation of radius error and node efficiency (corr = –0.181, *p* = 0.019) in RPFC. **(F)** Correlation of radius error and node local efficiency (corr = –0.103, *p* = 0.185) in RPFC.

As illustrated in Figure 8, only significant differences in clustering coefficient (*p* = 0.018), node efficiency (*p* = 0.019), and node-local efficiency (*p* = 0.012) among the right prefrontal cortex between AP and IP movements are observed, also implying that more functional connectivity is required in the right prefrontal cortex in in-phase movements. In addition, there exist a weakly positive correlation between radius standard deviation and network property from the right motor cortex, while only node efficiency of the right prefrontal cortex is weakly negatively correlated with radius error (Figure 9).

## Discussion

This study found that there is a weak negative correlation between the Δhbo and radius error in unilateral movement, implying that the enhancement of brain activity may benefit motor performance improvement and motion error reduction. Moreover, the brain network connectivity indicator of the anti-phase task is smaller than that of the in-phase task in bilateral movement, which is consistent with the observation of the worse kinematic performance in AP movements than in IP movements. This finding suggests that coordination performance might be correlated with brain activity, such as the connections of various brain regions. These results indicate the important meaning of the impact of neural activity on the motor.

### Unilateral movements

Figure 3 revealed the better performance of the dominant hand than that of the non-dominant hand in radius error (Figure 3C) and radius standard deviation (Figure 3D) when performing the unilateral movement. This finding might be attributed to the task difficulty relative to the non-dominant hand and the routine usage in the dominant hand (such as writing and eating). Jancke and Shih also found that the dominant hand had better performance in radius variability (Jancke et al., 1998; Shih et al., 2021). Moreover, angular velocity error along clockwise is significantly higher than that of counterclockwise for the unilateral motion of a single hand (Figure 3A), owing to the possible faster motion of the clockwise than the counterclockwise, despite asking the participants to keep the same speed and following the guide circle. Lalonde also finds that the higher the walking speed, larger the walking error (Lalonde-Parsi and Lamontagne, 2015). The inverse relationship between speed and accuracy is the trade-off which is the aspect of skilled motion performance (Dexheimer and Sainburg, 2021). It existed in many situations such as baseball throwing (Freeston et al., 2015) and dart throwing (Juras and Słomka, 2013). Other results such as the angular velocity standard deviation, radius error, and radius standard deviation show no difference in motion direction. Similarly, there was no significant difference between the young in radial variability in Shih’s study (Shih et al., 2021). However, a significant difference in kinematic indicators, including radius variability and inter-limb synchronization in the elderly group existed in their research which pointed out that the elderly and disease people could be collected to get further finding. Furthermore, Figure 4 shows the activation of the contralateral brain regions, that is, the activation of the right brain region for left-hand movement and the left brain region for right-hand movement. Our results verified the results obtained in previous studies (Nishiyori et al., 2016) and also points out the location of the brain area for the calculation of blood oxygen activation and motion error. In addition, the finding in Figure 5 revealed that a weak negative correlation between the Δhbo and radius error gives evidence of a relationship between brain activity and motor control ability. It meant that accurate action control requires enough blood support in the brain, signifying that sufficient brain activation contributes to kinematic performance and complex action control. Moreover, Tan also found a negative correlation between angular error and amplitude of the β (13–30 Hz) event-related synchronization in an EEG study (Tan et al., 2014). Similarly, the activity of the neurons increased a significant reduction in the absolute error in dart-throwing (Khanjari et al., 2022). This is an interesting finding since the comprehension of the motor performance in neuron activity provides a measurement method for designing recovery projects that improve motion performance.

### Bilateral movements

Figure 6 shows that the limb control ability of the non-dominant hand was poor compared to the dominant hand during AP and IP movements. Since previous literature reveals that the asymmetric causal relationship from left M1 to right M1 might represent crosstalk at the cortical level, contributing to the stability of symmetrical bimanual movements (Maki et al., 2008), we conjecture that these control ability differences might be due to differences in brain network connections. On the other hand, Figure 7 shows that IP movements were more dependent on the dominant left cortical control, with more activation on the dominant hemisphere than the non-dominant hemisphere, while there was the same activation in both hemispheres during AP movements. Interestingly, where one hemisphere was more dominant, coordination between limbs in AP movements would be affected (Shih et al., 2021), which also illustrates our finding (Figures 6C,D) that the significant difference between non-dominant hand and dominant hand in radius and standard deviation. These observations are also consistent with the previous findings about the differences in brain activation between IP and AP tasks (Chen et al., 2005). However, the left hemisphere dominance implies that crosstalk or signal gating occurred at the transcallosal level during AP movement (Maki et al., 2008), which might also be one of the possible reasons for the worse coordination performance in AP movements. In addition, Maki and Wong also found a significant increase in left motor cortex activity in AP movement (Maki et al., 2008). Previous paired TMS studies have shown that the motor cortex has coherent inter-hemispheric faciliatory effects (Ugawa et al., 1993) and inhibitory effects (Ferbert et al., 1992), which work *via* the corpus callosum (Di Lazzaro et al., 1998). Moreover, previous structural equation modeling studies showed that the coupling of the left primary motor cortex to the right MI, the connections of two (left and right caudal dorsal anterior motor areas) PM to two MIs (left and right primary motor cortex) with negative interactions from left PM to right PM, and the functional influence from the SMA (supplementary motor cortex) to the right MI and right PM can contribute to bilateral coordination (Zhuang et al., 2005). These findings in brain connections might also explain the worse performance of the non-dominant hand than the dominant hand in AP movements, as shown in Figure 6.

Figure 8 shows a higher value in clustering coefficient, node efficiency, and node-local efficiency of IP movement than AP movement. Together with the typical increase in Δhbo with IP tasks and more pronounced activation at M1 than in other regions, these findings might answer the reason that AP movements require higher force output than IP movements (Tettamanti et al., 2013). These results infer that there are differences in neural activity and motor performance between the AP and IP movements. It could be used in the classification of brain-computer interfaces or provide the target of the non-invasive neuron stimulation treatment for stroke patients such as TMS and so on.

### Brain network analysis

We analyze the brain network activation and compare the correlation between the network indicators and kinematic parameters to further search the relationship between brain connectivity and motor control ability. Correlation values were used to construct brain networks in previous studies, and global and local indicators were calculated on the network (Mukli et al., 2021; Xu et al., 2022). However, the network constructed by correlation values can only focus on the linear relationship between the two channels, while PLV can study the phase synchronization of non-linear and non-stationary signals, thereby discovering more inter-brain correlations. In this study, there is a weakly positive correlation between network indicators and kinematic indicators in RMC (Figures 9A–C), which might illustrate motor performance inhabitation in non-dominant brain regions and be related to the performance of competitive effects of motor activities. Moreover, there was a weakly negative correlation in the RPFC (Figures 9D–F), which might explain that cognitive ability promotes kinematic performance. Previous TMS study has also found that corticocortical paired associative stimulation (cc-PAS) in the corpus callosum in the left and right hemispheres induced associations in connectivity between targeted cortical regions (Rizzo et al., 2009). These findings suggested that bilateral coordinated movements can evoke brain activation and contribute to motor cortex reorganization (Li et al., 2013; Han and Kim, 2016; Wu et al., 2020) and the movement becomes more stable as the activation of the brain region becomes stronger. By contrast, higher functional connectivity (including the higher average degree of connectivity, connection strength, network density, and efficiency) was associated with bilateral coordination, independent of task difficulty (Heitger et al., 2013). TMS-EEG studies have shown that cerebellar-induced prefrontal synchronization promotes working memory, but bout motor activity produces a competing effect (Du et al., 2018) that possibly explains the worse motion performance with more network connectivity in RMC. Fu and coworkers extracted brain network features of stroke survivors for kinematic comparison and they demonstrated that the connection between the brain regions presents the feature of brain activity, and those coordinated movements of lower limbs can help stroke patients recover (Fu et al., 2021). Therefore, coordinated movement is an important means to assess the level of rehabilitation. While exercising the patient’s athletic ability, it can stimulate the corresponding brain motor control areas to improve the effectiveness of rehabilitation training. In addition, coordinated movement-assisted rehabilitation protocols have proved that active resistance tasks are more difficult than active assist tasks, and they can provide strength training for bimanual patterns (Li et al., 2014; Berger et al., 2018). And our findings on the difference in brain activity in eight movements also give a choice to control the rehabilitation device or brain-computer interface.

### Limitations

There were several limitations that need to be noted in this study. First, only young healthy people were recruited and the findings may not be applied to aging people and neurological patients such as stroke directly (Xia et al., 2022). Zhang and coworkers found that old and younger groups showed a wide range of bilateral activation in the motor cortex (Zhang et al., 2021). Future studies will consider recruiting stroke survivors and age-gender-matched control subjects to compare the motor control ability (e.g., the radius variability or the phase synchronization) during coordinated movement. Second, the kinetic data only have the finger trajectories, and further study would include the EMG on the muscle when performing the movement tasks which could provide more information between the central and peripheral connectivity (Zhang et al., 2022).

## Conclusion

In summary, this study verified the activation of the contralateral brain region of one hand, the activation of the dominant brain region of the same movement, and the activation of the bilateral brain region of the opposite movement. Further network connection analysis found that both the right prefrontal cortex and right motor cortex influence accurate motion control, which verifies the control function in the non-dominant brain area on the other hand. This finding provided evidence for further exploration of central nervous system activity and peripheral motor capacity and could guide clinical rehabilitation of patients with impaired motor function.

## Data availability statement

The original contributions presented in this study are included in the article/supplementary material, further inquiries can be directed to the corresponding authors.

## Ethics statement

The studies involving human participants were reviewed and approved by the Ethics Committee of Northwestern Polytechnical University. The patients/participants provided their written informed consent to participate in this study.

## Author contributions

GZ, QH, and LL conceived and designed the study, made contributions to experiments, and reviewed and edited the manuscript. YC, XW, and HW performed the experiments and collected data. GZ, YC, and LL analyzed the data. YC and LL wrote the manuscript. All authors read and approved the final manuscript.
